# Artificial Intelligence as a Personal Coach: A Narrative Review of Benefits and Risks in Educational and Health Contexts

**DOI:** 10.3390/bs16081431

**Published:** 2026-08-19

**Authors:** Jason T. Potel, Madoka Kumashiro

**Affiliations:** Department of Psychology and Neuroscience, Goldsmiths, University of London, London SE14 6NW, UK

**Keywords:** artificial intelligence, coaching, personal development, education, health/fitness

## Abstract

The last decade has seen a rapid increase in individuals turning to artificial intelligence (AI) for advice related to their personal development, especially with the introduction of general-purpose large language models (LLMs) to the general public in 2022. This narrative review examines the potential benefits and risks of using AI for coaching purposes in educational and health contexts. Given that empirical studies on using general-purpose LLMs in these domains remain limited, this paper first synthesizes findings from purpose-built coaching chatbots designed to perform specific tasks that facilitate personal development in these domains and discusses limitations associated with studies that use older versions of chatbots. The reviewed evidence suggests that purpose-built chatbot coaching systems may have some benefits as they are generally well received and can support short-term motivation and selected behavior change, but effects for sustained, meaningful outcomes are inconsistent. We then reflect on the potential risks of using general-purpose LLMs as a coach without appropriate human oversight, by reviewing features of general-purpose LLMs, such as sycophancy, accuracy, and problematic patterns of use. Synthesizing these findings, we consider their implications before identifying potential directions for future research.

## 1. Introduction

This paper presents a narrative review of potential benefits and risks of using AI as a personal coach in educational and health contexts. This is important, as the rapid proliferation of artificial intelligence (AI) in recent years has transformed many users’ expectations about the powerful role technology can play in facilitating self-development. Since advanced general-purpose large language models (LLMs), such as ChatGPT, became publicly available in late 2022, users have increasingly turned to them, not only for quick information, but for guidance, motivation, reflection, and some form of self-improvement. In other words, they are turning to general-purpose AI to act as a coach, even though these systems were not primarily developed as coach systems. Accordingly, some AI companies caution users that responses may not be accurate and should be checked against professional advice, particularly for health-related questions (e.g., Anthropic, n.d.; OpenAI, n.d.).

Indeed, while the term “AI coach” has been used loosely in the public discourse and news media to describe a wide variety of instances of people looking to AI for guidance (e.g., Boyle, 2026; Chamorro-Premuzic, 2026; Sloat, 2026; Smith & Rickard, 2025), the academic literature has generally adopted a more limited definition of coaching. Hardly any empirical studies have examined the effects of individuals turning to general-purpose LLMs for advice, and existing findings may quickly become outdated due to the rapid pace of technological change and adoption (Z. Huang et al., 2025; Luo et al., 2025; Schimpf et al., 2026). Given that over a third of the population in OECD member countries used generative AI in 2025, with numbers expected to grow substantially (OECD, 2025, 2026), there is a pressing need for more research on potential benefits and risks of using AI as a personal coach.

The gap between widespread public use and empirical research is especially noticeable in educational and health contexts, even as more users turn to general-purpose LLMs like ChatGPT for practical guidance in these areas (working paper by the National Bureau of Economic Research, Chatterji et al., 2025). These domains are particularly relevant for goal-directed behaviors in personal development but have produced relatively fewer reviews compared to the effects of AI coaching in other domains, such as the workplace (de Haan et al., 2026; Plotkina & Sri Ramalu, 2024; Terblanche et al., 2024). The reviews already published (e.g., Oh et al., 2021; Singh et al., 2023) covering these two contexts do not pay sufficient attention to the potential risks and unintended consequences, instead focusing almost entirely on potential benefits and user interest. Even more recent reviews produced since the popularization of ChatGPT and other LLMs (Guan et al., 2025; W. Huang et al., 2025) consistently approach the technology with a general optimism without considering the developing risks associated with factors like sycophancy and emotional dependence. These reviews have primarily evaluated purpose-built AI coaching systems and user outcomes, but they have given less attention to whether such findings can be applied to unsupervised use of general-purpose LLMs and their potential risks.

This narrative review synthesizes research findings on AI coaching in health and educational contexts by analyzing their benefits, limitations, and risks, before considering implications for future research. As there is limited direct empirical evidence on using general-purpose LLMs for “coaching” advice in health and educational contexts, the current narrative review first seeks to synthesize selected findings from studies using purpose-built and pre-AI coaching chatbots to examine evidence regarding the effectiveness of using purpose-built AI and non-AI chatbots in coaching. We then use this foundation to evaluate the benefits and risks of applying such findings to the general-purpose LLM chatbots (e.g., ChatGPT, Claude, Google Gemini, etc.) for personal development guidance in these contexts.

We thus approach this review with five objectives: (1) examine the benefits of integrating AI into personal coaching; (2) summarize empirical research on purpose-built AI coaching systems; (3) identify the limitations of the evidence; (4) consider whether findings can be realistically applied to general-purpose LLMs by examining the relevant literature; (5) synthesize the implications and recommend further investigation into general-purpose LLM coaching as its usage becomes more widespread.

We selected empirical studies for their relevance to AI-based coaching, particularly in educational and health/fitness contexts, and also reviewed studies that discuss features of general-purpose LLMs that may pose risks for using such LLMs to seek out advice in these domains. This narrative review is intended to produce a conceptual synthesis of the current literature rather than a systematic or scoping review, and does not follow a fixed protocol for search, screening, or quality appraisal. Due to the literature spanning multiple disciplines and different AI systems over time, we selected studies that directly examined AI systems performing coaching-relevant functions (e.g., goal-setting, feedback, motivation, reflection, etc.) rather than an exhaustive, single-database search. Relevant literature was identified by performing targeted searches through databases including Google Scholar and PubMed, using combinations of keywords associated with artificial intelligence, coaching, personal growth, chatbots, education, health, well-being, and fitness. Searches focused primarily on work published within the last five years, given the technology’s rapid evolution, with the search used to identify the primary empirical AI-coaching studies synthesized in Section 4 and Section 5 completed on 30 April 2026.

## 2. Defining Terms: Coaching and AI Systems

### 2.1. Coaching

For the purpose of this paper, coaching is understood as a supportive process that helps individuals develop their maximum potential and move toward meaningful goals (e.g., Bachkirova et al., 2023; Whitmore, 1992). This definition is consistent with the broader literature on psychological and organizational coaching, which typically emphasizes goal-setting, reflection, goal-directed change, and the individual’s active role in their personal development (e.g., Grant, 2013). In operational terms, we treat AI systems as performing a coaching function when, rather than simply answering factual queries, the interaction supports one or more of the following: goal clarification, self-reflection, feedback, action planning, motivation, or behavior change.

Such coaching functions may contribute to personal development, and potentially to personal growth, which is understood as continued development and increasing self-knowledge and effectiveness (Ryff, 1995). For the purposes of this review, personal development is used as a broad term referring to intentional changes through which individuals acquire knowledge, skills, self-understanding, healthier habits, or a greater capacity to pursue meaningful goals. It therefore encompasses the range of proximal outcomes examined in the studies reviewed here, including motivation, self-reflection, educational performance, health behavior, and self-regulation.

Although our definition may differ from the typical definition of a more structured, goal-directed process involving a collaborative partnership between a trained coach and a client, there is also no agreed definition in the various fields of psychological and organizational coaching (e.g., Bachkirova et al., 2023; Passmore & Lai, 2020). We keep the focus on such agreed commonalities between most definitions on individuals trying to maximize their potential, as the empirical literature has so far not kept up with the rapid adaptation of general-purpose LLMs for self-directed personal development opportunities, without the involvement of a human coach.

Interestingly, the word coaching was derived around 1830 from a coach carriage to initially describe Oxford University tutors who help “carry” their students through exams (Bachkirova et al., 2023). Since then, the term has been used in a wide variety of contexts, including in educational, health, sports and other hobbies and pastimes, lifestyle, and workplace settings. In non-academic contexts, the term AI coach seems to refer to offering guidance on any topic, including being accused of acting as a “suicide coach” in a series of lawsuits reported in news articles (e.g., Betts, 2025), suggesting the need to also consider coaching in broader contexts, including when it moves away from the intended positive focus on personal development.

So how might AI serve as a coach to support personal development, especially in the context of educational and health domains? AI systems may be able to help users with the common functions of a coach (e.g., clarify and set goals, facilitate self-reflection, offer feedback, and sustain behavioral change) and offer such support and guidance in a non-directive manner that helps individuals retain their sense of autonomy (Ryan & Deci, 2019). Thus, this approach also aligns with self-determination theory (Ryan & Deci, 2000), which posits that individuals are more likely to become intrinsically motivated to work toward their personal growth goals when their needs for autonomy, competence, and relatedness are fulfilled.

### 2.2. AI Systems

We now distinguish between the various types of technology discussed throughout this paper^1^, summarized in Table 1. A chatbot is “a computer program that simulates human conversation with an end user” (IBM, 2021), a category of tools that has existed for roughly 60 years with a wide range of sophistication. Early systems such as ELIZA relied on scripted pattern matching rather than learned language capabilities (Weizenbaum, 1966). More sophisticated chatbots incorporate artificial intelligence (AI), a concept that broadly describes the “capability of non-human machines… to perform, task solve, communicate, interact, and act logically as it occurs with biological humans” (Gil de Zúñiga et al., 2024, p. 1).

Natural language understanding (NLU) is a subfield of the broader discipline of natural language processing (NLP), which is concerned more generally with enabling computers to process and generate human language; NLU specifically refers to the capability of extracting meaning and intent from text or speech, which is the function most relevant to how task-oriented chatbots identify what a user wants (Adamopoulou & Moussiades, 2020). Machine learning (ML) refers broadly to computational methods that learn patterns from data rather than relying solely on explicitly programmed rules (Adamopoulou & Moussiades, 2020). Chatbots that use NLU will typically incorporate either rules-based or ML-based approach, if not a combination of the two. Some studies use the broader term NLP when describing systems that appear to perform intent-recognition functions. In the text and tables, we retain the terminology used by the original authors where possible and indicate the apparent functional category when this can be determined from the study description.

Finally, large language models (LLMs) represent the most recent development within NLP: trained on extensive collections of text, they generate language in response to user input rather than relying solely on predefined intents and responses, allowing them to simulate general conversation across a wide range of purposes (Cloudflare, n.d.). Unlike task-specific chatbots, these systems are not restricted to a predefined domain, although an LLM may be adapted or constrained for a particular coaching, educational, or health application.

Any of these AI systems can be purpose-built (i.e., explicitly designed to perform a specific task). However, some general-purpose LLMs (including ChatGPT, Claude, and Gemini) are trained to be used across a variety of services. In addition to the type of programming (LLM vs. rules and/or ML-based models), how these systems are programmed (purpose-built versus general-purpose) and how they are used (alone versus in coordination with human coaches) may further shape the user’s experiences and outcomes.

An important factor in research on AI coaches is whether users interact with an AI chatbot specifically programmed to provide a coaching service or with a general-purpose LLM (such as ChatGPT, Claude, DeepSeek, Gemini, and Grok). Although general-purpose LLMs have been shown to support personal development like learning a new language, a majority of these studies did not utilize the technology for coaching purposes (G. Liu & Ma, 2024). Limited research on this may be due to the technology’s recency: general-purpose LLMs became widely available to the general public toward the end of 2022. While these early models were often associated with frustrating experiences or were faulty in function, the rapid advancement and constant introduction of more powerful models make it difficult for research findings to keep pace (Z. Huang et al., 2025; Luo et al., 2025; Schimpf et al., 2026).

General-purpose LLMs may also be more associated with risks that we will discuss later, as they are not programmed with a specific area of behavioral change in mind. Given that the majority of research on coaching is based on purpose-built coaching chatbots, this paper will mainly focus on this category, although we will discuss the risks associated with general-purpose LLMs in a later section. In this paper, we generally refer to AI chatbots, AI coaching and AI software, including LLMs and task-specific NLU systems as AI, unless we note that the technology was not based on AI.

## 3. Benefits of Integrating Technology into Coaching

Before we delve into the empirical literature on AI-based coaching, it is useful to consider earlier research on technology-assisted coaching. Studies using pre-AI systems provide a foundation for understanding how digital tools can support behavior change and personal development. For example, a Danish experiment using a non-AI robot coach that could recite prewritten lines and perform pre-choreographed gestures found that participants felt more confident about retaining behavioral change on their chosen goal, compared to the control participants, especially if the robot coached with more emotions (Jelínek & Fischer, 2021). A 2-week Korean study indicated that keeping a non-AI Diarybot programmed with pre-scripted responses triggered by the user’s journal entry can stimulate self-reflection, which may help support meaningful personal development (Park et al., 2021). In another four-week experiment, eight sessions of pre-scripted non-AI chatbot-based coaching incorporating lessons on a variety of life skills (e.g., emotional self-awareness, conflict resolution, assertive communication, etc.) were found to be valuable for adolescents, who generally described it as useful, innovative, and easy to use (Gabrielli et al., 2020).

Thus, the findings of these early, non-AI-based coaching sessions suggest that AI chatbots may also be able to serve some of the core functions of a coach, as users found early pre-scripted chatbots and robots to be helpful, reporting boosts in their well-being and confidence levels in tackling their goals, suggesting that more advanced AI systems could potentially be more effective. Certain features of AI- and other technology-based coaching may, in some contexts, offer advantages over their human counterparts. Some of the largest barriers to effective coaching for human coaches have to do with lack of time and financial resources to access coaching, as well as other negative factors directly related to the coach, such as inexperience, lack of knowledge or expertise, feeling overworked, or feeling negatively affected by the coaching process (Schermuly & Graßmann, 2019). Unlike human coaches, chatbots are always accessible, not distracted by personal issues, and many LLM-based chatbots or coaching-related software are free and easy to access or are relatively inexpensive (Arakawa & Yakura, 2024).

As a non-clinical activity focused on goal achievement, coaching does not usually involve the breadth of interpersonal training found in professions that diagnose and treat pathologies (e.g., therapist or social worker; Grant, 2006). Accordingly, it may be easier to delegate some of the coaching functions to automation. For example, AI chatbots can quickly deliver introductory guidance to get started on a new task or pursuit (Afzaal et al., 2025; Loughnane et al., 2025; Yin et al., 2024). Thus, engaging and motivating users to attempt forays into new areas and activities may represent one potentially useful application of AI coaching.

Further, AI’s ability to endlessly generate bespoke responses that cater to each individual’s unique set of needs could grant users greater motivation to complete tasks in their everyday lives. Some new models of AI chatbots have been programmed with emotional intelligence aimed at identifying and reacting to the user’s emotional state (Yin et al., 2024). In human coach-client relationships, high quality relationships have been found to lead to fewer negative outcomes of coaching (Schermuly & Graßmann, 2019), and users may similarly benefit from having a responsive AI chatbot coach. Nevertheless, another study (Grant, 2014) comparing different factors of success in human coaching found that satisfaction with the coaching relationship does not actually predict successful coaching outcomes, but having a coaching relationship that was similar to an ‘ideal’ coach was, alongside receiving autonomy support. Interestingly, goal-focused relationships led to best client outcomes 12 weeks later, which bodes well for AI coaching, especially for AI systems that are specifically programmed for coaching purposes.

Thus, AI may combine the features from two distinct approaches which may potentially explain why some users experience AI coaching as beneficial. First, due to their interactive nature, AI coaches can provide metacognitive support tailored to user responses. Multiple studies have found that this allows users to direct AI chatbot responses (Fernando et al., 2024; Wei et al., 2022), which could simulate an ideal coaching relationship that provides users with feelings of autonomy. Second, along with this responsive, social element, AI systems offer a comprehensive delivery of knowledge, scaffolding information into digestible and manageable packages, which may help users develop and refine new habits and skills (W. Huang et al., 2025). Nevertheless, some of these helpful features also contribute to risks in using AI chatbots, especially general-purpose LLMs as a coach, which we will discuss later.

We now turn to reviewing the empirical literature that has examined purpose-built AI coaching systems in educational and health contexts. Table 2 organizes the evidence discussed in Section 4 and Section 5 by theme and summarizes the overall pattern of findings. While randomized controlled trials (RCTs) generally provide stronger evidence than nonrandomized or uncontrolled designs, much of the evidence reviewed in the next two sections remains preliminary, including pilot and feasibility studies, and several studies use uncontrolled designs; findings from these studies should be interpreted cautiously. A more detailed summary of the selected primary empirical AI-coaching studies discussed in Section 4 and Section 5 is provided in Supplementary Table S1.

## 4. Educational Settings

In this section, we selectively consider the empirical evidence for using purpose-built AI chatbots in educational settings in terms of user experience and engagement, followed by educational performance. Recent reviews have generally reported promising findings, particularly regarding motivation for self-regulated learning (Guan et al., 2025; W. Huang et al., 2025).

Studies relying mainly on self-report data generally indicated positive initial user experiences and engagement after interacting with purpose-built chatbots. In one study, educational chatbots with even very limited AI capabilities were associated with more enjoyable learning for students (Chuang et al., 2023). A German Wizard of Oz study, which did not actually feature AI, suggested that students can build a rapport with a coaching interface presented as AI and use it to reflect on exam anxiety (Mai et al., 2021). A generally positive attitude from students incorporating AI NLP chatbots into debate preparation was also reported, such as the chatbots helping stimulate new thoughts, introduce fresh approaches, and induce calming and productive emotions (Yin et al., 2024). A study involving a short follow-up also suggested that such effects may last longer. Interactions with scripted AI chatbots were associated with improvements in physical, social, and academic well-being among high school students a week later (Sia et al., 2021). Nevertheless, these were all one-off or short-term studies and did not establish whether the reported effects were sustained over longer periods.

Findings were more mixed for studies with repeated engagement with AI chatbots. For example, in a month-long study integrating chatbots into a Singaporean chemistry curriculum, some students expressed frustration over the virtual coach giving incorrect hints or misunderstanding the questions (Jasin et al., 2023), which is a common experience, especially with LLM-based chatbots, that will be discussed in a later section. In a year-long experiment, AI chatbots for writing skills feedback (Feedbot) and reading support (Litbot) received mixed reactions from participants who largely refrained from fully exploring the former’s functions (with some requesting a human coach), while criticizing the latter’s generic responses, which could not directly address the questions prompting them (Neumann et al., 2021). Further, while students rated purpose-built chatbots based on a general-purpose LLM high in perceived usefulness and ease of use, they still preferred human guides (Neumann et al., 2025). Although AI chatbot-based language learning initially showed positive student engagement, this interest quickly dropped after use compared to a human partner, suggesting that the early interest may be due to the ‘novelty’ effect, or how users initially respond more positively to new experiences (Fryer et al., 2017). These findings indicate that initial acceptability does not necessarily translate into sustained engagement.

Findings for educational performance were also mixed. On the positive side, in one five-week study, students who engaged with a pre-programmed AI chatbot that asked questions and provided feedback and explanations showed improvements in their performance and higher levels of intrinsic motivation, although the chatbot did not provide a significant advantage over peer feedback (Fidan & Gencel, 2022). Moreover, math students learned just as much from a session with an AI rules-based chatbot as they did from a session with the global educational non-profit Khan Academy with around 40% even preferring the automated interaction (Cai et al., 2021). These findings suggest that some purpose-built chatbots may support motivation or performance on structured tasks, sometimes producing outcomes comparable to those of established digital learning resources.

While encouraging, findings from other studies paint a more neutral or negative picture, with many showing no benefits or worse outcomes from using AI coaches. For example, in another short-term study, nursing students training with an AI chatbot coach did not show statistically significant differences in knowledge, clinical reasoning competency, or confidence compared with the control group, suggesting negligible performance benefits (Han et al., 2022). Over the course of five two-hour class sessions, programming students only performed slightly better when using AI chatbots than when relying on traditional educational approaches; however, they performed best when guided by digital technology that did not include AI features (e.g., videos and PowerPoint slides; Ait Baha et al., 2023). When used for a children’s story time session, AI chatbots proved no better at assisting with reading comprehension skill-building and even demonstrated lower productivity, lexical diversity, and topical relevance compared to control groups (Xu et al., 2021). Thus, these studies do not support a general performance advantage for AI coaching over comparison conditions.

Together, these findings suggest that the effects of AI coaching in educational contexts are mixed: while it may be generally well received and effective in the short term for structured tasks, evidence for more performance related outcomes and sustained motivation is inconsistent, with users showing preference for human interactions in certain contexts.

## 5. Health Settings

In this section, we selectively consider empirical evidence for benefits of purpose-built AI systems in the health, well-being, and fitness domains, in terms of psychological outcomes and conversational processes, health behavior change, and sustained or clinically meaningful outcomes. The findings show a similarly mixed pattern but with somewhat more consistently positive findings for short-term health behavior change.

Findings from studies examining psychological outcomes and conversational processes suggest that AI coaching may support some of the underlying mechanisms of effective coaching. For example, a simple automated speech-based chatbot using limited NLU capability, which may be nominally considered AI, was associated with higher levels of self-reported personal-growth directed behaviors and well-being levels in adults over the course of three weeks (Aymerich-Franch & Ferrer, 2022). Further, AI chatbots may be able to offer autonomy support which is key to preserving autonomous (vs. controlled) motivation that drives personal growth motives, in line with self-determination theory (Hagger & Star, 2026; Ryan & Deci, 2000). A recent analysis of 4352 messages exchanged over one week between 38 university students and a purpose-built LLM-based well-being coach identified agency- and autonomy-related behaviors in interactions involving 97.4% of participants; these behaviors accounted for nearly one-third of user utterances (Shah et al., 2026). However, this study examined conversational content rather than changes in participants’ autonomy or well-being and thus does not establish that interaction with the AI coach produced downstream psychological benefits.

Findings for health behavior change were more consistently positive. A 5-week pilot study recruiting 42 participants found that the use of conversational AI chatbots correlated with increased physical activity compared to non-conversational or non-AI control groups (Hassoon et al., 2021). Similarly, even a simple rules-based AI “digital coach” significantly increased participants’ physical activity in a 10-week pilot study, with more modest improvements in their mental well-being and self-efficacy; however, these effects were not sustained during autonomous use (Santini et al., 2023). A six-week study using more sophisticated chatbots was also associated with an increase in users’ physical activity by over two and a half hours a week (To et al., 2021). A pilot study incorporating an AI chatbot into virtual health coaching was associated with participants increasing physical activity by an average of nearly 110 min over the course of 12 weeks while also increasing Mediterranean diet scores from 3.8 to 9.6 (Maher et al., 2020).

Other findings suggest gains in healthy eating: one pilot study indicated that using a healthbot with primitive NLP properties was associated with slight increases in users’ fruit and vegetable intake and decreases in consumption of sweet beverages, fried foods, and snacks over the course of four weeks (Dhinagaran et al., 2021). In a one-week weight management pilot study with over 200 participants, use of a slightly more complex AI chatbot was associated with significant improvements in overeating habits, snacking habits, self-regulation of eating behavior, depression, and physical activity (Chew et al., 2024). Taken together, these studies suggest that AI coaching may be able to support short- to medium-term improvements in physical activity and diet, though several relied on small samples and pilot-scale designs.

Fewer studies examined clinically anchored outcomes or monitored participants over a longer time period, and here the findings were more mixed. Interaction with NLU mental health chatbots over one month correlated with a significant reduction in stress, anxiety, and depression; in fact, roughly half of the participants who began the study with clinical symptoms ended the study a month later with their symptoms no longer flagging clinical concern (Daley et al., 2020). However, the lack of a control group means that these changes cannot be specifically attributed to the use of an AI chatbot. In contrast, when used to counsel dieting children in a long-term randomized controlled trial, incorporating AI did not improve sustained weight loss, although there were improvements in other physical outcomes (Stasinaki et al., 2021). Together, these two studies suggest that even where AI coaching is associated with sustained improvement in some outcomes or for some population, this does not guarantee sustained improvement across all outcomes of interest or in other populations.

These patterns are broadly echoed in existing review papers. A recent scoping review of 33 studies on AI chatbots and healthy behaviors found that 81.67% of comparisons showed positive outcomes with benefits primarily including physical activity, smoking cessation, stress management, and diet (Fu et al., 2026). However, no definitive conclusions could be derived from a systematic review of 9 papers examining AI chatbots used to promote physical activity regarding the efficacy of chatbot interventions on physical activity, diet, and weight management/loss (Oh et al., 2021). Another systematic review of 31 studies on AI conversational healthcare agents designed in part to support behavioral change found generally mixed user-perceptions in analyzing qualitative reports (Milne-Ives et al., 2020).

Taken together, these findings suggest that using purpose-built AI chatbots in health settings may yield benefits for many users, especially in the short term for behavior change and other self-reported outcomes, but the evidence for sustained or clinically meaningful outcomes is mixed. The studies reviewed so far also have limitations that we discuss in the next section, and the evidence discussed also does not establish that findings from purpose-built health chatbots can be generalized directly to unsupervised use of general-purpose LLMs.

## 6. Limitations of Purpose-Built AI Coaching Systems

In general, the evidence reviewed thus far paints a nuanced picture of the effectiveness of using AI systems as a guiding coach in educational and health contexts. We next discuss several important limitations of studies that use purpose-built AI systems for coaching purposes. First, this paper functions as a selective narrative synthesis rather than a systematic review of the current state of research into AI coaching and therefore does not aim to provide an exhaustive account of the literature. In addition, many studies are based on small sample sizes, short study durations, and self-report, limiting the ability to draw conclusions about long-term effectiveness and actual, observable sustained behavioral change (Hassoon et al., 2021; Milne-Ives et al., 2020). There is also widespread variability in the types of technology used, which may make it difficult to compare findings across studies or to determine which specific features of AI systems may be driving the observed effects.

Moreover, the ‘novelty effect’ observed in using chatbots (Fryer et al., 2017), where initial engagement quickly fades, likely extends to other AI-based systems and the studies reviewed here. In general, technology’s rapid evolution has accelerated the cycle of cultural trends and constant introduction of new gadgets, suggesting that coaching devices may also lose engagement as novelty wanes. Beyond novelty, engagement in these studies could depend on general attitudes toward AI. For example, trust in online content, life satisfaction, and general optimism are primary predictors of trust in AI (Yankouskaya et al., 2026). Some studies find that women are more likely to show a greater resistance to AI than men, particularly when it comes to delegating labor across education and health (Møgelvang et al., 2024; Nazaretsky et al., 2025; Russo et al., 2025), and there could also be cultural variations, with some studies finding that citizens from certain regions (e.g., Africa, Asia, and the Middle East) exhibit more trust and willingness to integrate AI (Yankouskaya et al., 2026). Nevertheless, given that retention rates tend to be low in studies involving AI healthcare interventions (e.g., Jabir et al., 2024; Milne-Ives et al., 2020), it may be difficult to separate out resistance to technology vs. general resistance to behavioral change, unless more systematic longitudinal intervention studies are conducted comparing performance of AI systems to human coaches.

Relatedly, it should be noted that some of the studies reviewed are over five years old. While these still represent examples of AI, technology’s rapid advancement, particularly over the last five years, suggests that studies conducted even a couple of years ago may not accurately reflect the capabilities of current systems, particularly general-purpose LLMs that demonstrate substantially improved performance (Anh-Hoang et al., 2025; Lin et al., 2024). Nevertheless, the rapid pace of development complicates efforts to maintain an up-to-date and stable evidence base (Wang et al., 2025).

Finally, this review has primarily examined purpose-built coaching chatbots used in research, instead of the general-purpose LLMs that are increasingly being used as ‘coaches’ for personal development. As previously noted, the term “AI coach” has been applied broadly in public discourse and news media, including in harmful contexts (Betts, 2025; Boyle, 2026; Chamorro-Premuzic, 2026; Sloat, 2026; Smith & Rickard, 2025). However, the empirical research on users turning to general-purpose LLMs for coaching-related purposes remains scarce. We now discuss potential risks of using general-purpose LLMs as a coach, particularly in the absence of oversight from human coaches or experimenters.

## 7. Potential Risks

The evidence reviewed in the preceding sections centers on purpose-built coaching systems, which provide structured, task-specific AI support. General-purpose LLMs, increasingly used for the same purposes without that structure, introduce a distinct set of considerations. Despite its promise, use of AI, particularly general-purpose LLMs, as a coach introduces a range of risks that may undermine its effectiveness or negatively affect users. Several of these risks are linked to potential benefits discussed in Section 3 and are summarized together with their corresponding safeguards in Table 3. As direct empirical evidence on using general-purpose LLMs as coaching remains limited, some of the risks described below are drawn from emerging commentary, policy discussions, and media reports, in addition to direct empirical evidence.

First, one of the most important concerns is the tendency of general-purpose LLMs to produce agreeable or affirming responses that could often be excessive, a phenomenon described as sycophancy, which may affect perception-based decision-making (Cheng et al., 2026). In one empirical study (Cheng et al., 2026) analyzing 11 different LLMs, AI-generated responses affirmed users 49% more than human responses. The more sycophantic the AI responses, the higher participants rated them, leading to greater trust and likelihood of continued use. Even a single exposure to a sycophantic response was enough to make participants less willing to take responsibility for conflicts, while becoming more convinced that they were right. Although such sycophantic responses may enhance short-term satisfaction and engagement, effective coaching often requires asking challenging questions and providing uncomfortable pushback to facilitate personal development, which a chatbot may be less able to perform without human involvement (Arakawa & Yakura, 2024). Excessive affirmation, in addition to feeling validated and a lack of accountability, may reinforce existing attitudes and behaviors rather than motivate individuals to critically self-reflect and take meaningful actions toward change.

Moreover, such sycophantic responses may validate maladaptive thought processes and may also affirm and exacerbate symptoms of anxiety, depression, and other forms of mental illness. Conversational AI may be perceived as emotionally safe and readily available, which may encourage users to overdisclose personal details and engage in excessive rumination of negative thoughts. Viewpoint and commentary articles have raised concerns that such patterns could potentially exacerbate psychotic experiences in vulnerable individuals (Carlbring & Andersson, 2025; Hudon & Stip, 2025). Although extreme outcomes are likely rare, emerging concerns in the popular discourse (e.g., “suicide coach” lawsuits discussed in a newspaper; Betts, 2025) and AI’s ability to give guided instructions on any topic, however inappropriate, may lead to potential harm for self and others.

Even when consequences may not lead to severe harm, turning to AI for support and advice may contribute to problematic patterns of use. The accessibility, responsiveness, and perceived non-judgmental nature of conversational agents can encourage frequent and emotionally salient interactions (Xie et al., 2023). For some users, particularly those experiencing loneliness or distress, this may lead to increased reliance on AI systems (Xie et al., 2023). A longitudinal study suggests that lower social connection may predict subsequent increases in social-chatbot use, while greater use may predict increased emotional isolation, though the authors caution against strong conclusions (Folk & Dunn, 2026). Chatbots can thus lead to psychological dependence, which could potentially yield salience, tolerance and withdrawal, a marker for addiction (Xie et al., 2023).

Finally, AI coaching systems may be limited by issues of accuracy and reliability, and any attempt to treat AI as an expert in education or health should be met with proactive caution. As reported earlier, participants may complain of AI systems providing inaccurate information or misunderstanding inquiries (e.g., Jasin et al., 2023; Kurniawan et al., 2024). This is a common issue: for example, one recent investigation found that ChatGPT only achieved about 85% accuracy, occasionally changing its answers from correct to incorrect or vice versa upon receiving additional questions (Ding et al., 2023), while another observed it endorsing incorrect statements up to 26% of the time (Khatun & Brown, 2023). That said, it should be noted that these findings are task- and model-specific rather than general estimates of LLM accuracy. While AI systems, particularly general-purpose LLMs, still produce “hallucinations”, performance on some tasks has improved (Anh-Hoang et al., 2025; Lin et al., 2024; Wang et al., 2025).

As it currently stands, however, general-purpose LLMs are known to generate plausible but incorrect information (“hallucinations”), and their responses may vary depending on prompt phrasing or conversational context (Ding et al., 2023; Khatun & Brown, 2023; Lin et al., 2024). In one empirical study of LLM behavior in a medical-information context, general-purpose LLMs often prioritized being helpful and agreeable over being accurate, even complying with illogical requests despite having access to factual information (Chen et al., 2025). Even when general-purpose LLMs are accurately able to describe a medical condition in a stand-alone test, users interacting with the same LLMs performed no better than control participants at identifying relevant conditions or selecting appropriate courses of action (Bean et al., 2026). This suggests that strong stand-alone performance may not translate into effective guidance during real user interactions. In coaching contexts, such inaccuracies may be particularly problematic when users rely on AI for guidance in domains such as education or health, where incorrect advice may have tangible consequences (Milne-Ives et al., 2020; Oh et al., 2021).

These concerns also raise broader ethical and professional issues as reflected in both corporate and policy-oriented literature. For example, the World Health Organization (WHO) issued ethics and governance guidance on large multi-modal models in 2025, recommending transparency about system limitations and data practices, protection of autonomy and privacy, accountability, and human oversight (World Health Organization, 2025). Although providers of general-purpose LLMs warn that responses may be inaccurate and should not replace professional advice (Anthropic, n.d.; OpenAI, n.d.), warnings alone may not fully counteract users’ trust in the interaction, given that sycophantic responses have been found to be preferred and trusted (Cheng et al., 2026). In mental-health contexts, AI coaching should be clearly distinguished from counseling, psychotherapy, and clinical care and should not be presented as a substitute for qualified professionals. Additional safeguards include ensuring that AI systems respond appropriately to signs of vulnerability and recognize when users should be directed to professional support (Carlbring & Andersson, 2025; McBain et al., 2025).

Table 3 synthesizes the LLM characteristics, potential coaching value, and principal risk associated with each, and the safeguards suggested in the literature reviewed in this paper.

## 8. Implications and Future Directions

The evidence reviewed on AI coaching involving purpose-built AI chatbots in the educational and health domains suggests mixed effects. With its responsive programming and ability to provide information in a digestible manner, AI may be able to facilitate self-reflection, short-term motivation, and selected behavioral changes and help preserve autonomy needs, particularly in the health domain (W. Huang et al., 2025; Shah et al., 2026). However, our review also suggests that using AI chatbots may not result in sustained behavioral changes, improvement in outcomes, or user engagement, with users in some studies preferring human coaches or performing better with non-AI alternatives (Ait Baha et al., 2023; Neumann et al., 2025). More studies, especially experimental, longitudinal studies, and randomized controlled trials, are needed to understand the mechanisms of what makes an AI chatbot more effective, under what circumstances AI may be effective in providing a lasting change in users, and which users appear to benefit the most and least from AI coaching.

We focused on reviewing research with purpose-built coaching chatbots instead of the more advanced general-purpose LLMs that are increasingly being used for coaching purposes, as the rapid proliferation of this new technology means empirical research is lacking in this area. As more users start depending on general-purpose LLMs as a coach, without oversight from human professionals, researchers and policy makers should take note of the risks we identified that are associated with using general-purpose LLM chatbots as a coach. The same features that make turning to AI as a coach appealing, such as continuous availability, personalization, responsiveness, and sycophantic encouragement, may also contribute to dependence, reduced critical self-reflection, and misplaced trust in inaccurate information. Sycophantic responses may also encourage the pursuit of even negative and maladaptive beliefs and goals rather than encouraging constructive reflection (Cheng et al., 2026).

The risks associated with sycophantic responses are particularly important because effective coaching often involves constructive challenge, critical reflection, and accountability instead of being continually affirmed (e.g., Grant, 2014). Moreover, Mukherjee et al. (2024) suggest that while LLM chatbots might offer some mental health support, they lack the nuanced empathy required to function with necessary accuracy and ethics. It is particularly important that AI should be programmed to recognize and stop sycophantic encouragement when users engage in maladaptive and distressing thought processes (Chen et al., 2025; Cheng et al., 2026; Hudon & Stip, 2025), as such sycophantic responding could give rise to providing dangerous and harmful guidance, such as providing advice on suicide (subject of several lawsuits as mentioned earlier in a newspaper article; Betts, 2025). Given AI’s tendency to hallucinate, more safeguarding practices and public education measures are needed to inform users to be mindful of potential hallucinations, sycophantic responses and the possibility of developing unhealthy dependence on AI (Cheng et al., 2026; Lin et al., 2024).

Nevertheless, our review suggests that some features of AI could benefit the coaching process. Its ability to provide immediate feedback, offer tailored guidance, help with practical aspects of goals and provide structure, and offer warm, responsive, and interactive conversation suggests that AI may be able to help support important autonomy, competence, and relatedness needs (Ryan & Deci, 2000). AI coaching systems should therefore be designed to encourage user’s initiative and preserve their agency while providing warm, friendly and accurate advice, without promoting excessive affirmation or dependence. Thus, with appropriate professional oversight and better safeguards built into AI systems, along with more education to the general public about risks of using LLMs in this way, AI could prove to be a useful aid in the coaching process.

Design recommendations may need to be reconsidered and updated, as the technology and patterns of use evolve rapidly. For example, earlier research on a relatively limited NLP coach suggested increasing emotional engagement by further anthropomorphizing the AI (Yin et al., 2024). However, with the popularization of general-purpose LLMs, such attempts to increase emotional engagement may also increase the risk of unhealthy attachment, given that users can perceive AI as personal and trustworthy (Carlbring & Andersson, 2025). Recent findings suggest that such an emotionally responsive LLM-based coaching system should preserve user agency during emotional disclosure while recognizing and redirecting early negative conversational loops (Shah et al., 2026).

AI may be best used to complement or assist in human coaching practices. The use of AI technology in coaching practice is not necessarily meant to eliminate the human component and in fact can be designed to enhance its overall impact (Moreno-Blanco et al., 2021). Some of the studies employed hybrid approaches in which AI chatbots were introduced into human-supervised educational environments or integrated as a supplementary tool to be used in between routine sessions with human coaches (Arakawa & Yakura, 2024; Chiu et al., 2023). The findings suggest that using chatbots to assist human-led coaching was effective, especially when AI chatbots provided routine feedback, prompts, action planning and reflection between sessions, while human coaches retained responsibility for goal clarification, constructive challenge, and intervention. However, the findings were based on short-term processes and did not establish that hybrid coaching was more effective than AI or human only coaching.

A systematic review of 35 studies (Loughnane et al., 2025) comparing human, AI, and hybrid coaching within digital health interventions suggests that all three forms were found to be feasible and acceptable to users, with both human and AI coaching also associated with positive outcomes. Nevertheless, the authors also cautioned that more research on hybrid approaches is needed. A review of 43 papers on student motivation suggests that although chatbots were effective, they still fell behind interventions that involved human guidance and feedback (W. Huang et al., 2025). Together, these findings suggest that hybrid coaching is promising, but more research using experimental and longitudinal designs is needed into when and how to use AI effectively in coaching practices. In addition to other safeguarding matters already discussed, research may also be needed into how to instill awareness in users of general-purpose LLMs’ limitations and to encourage users to seek out human professionals for coaching matters.

Regarding potential avenues for future research, multiple studies on AI-human interaction emphasize the need for methodologies to include longitudinal designs to distinguish sustained change from plateauing or declining engagement as the novelty effect fades (Abdi et al., 2022; Han et al., 2022; Jasin et al., 2023). Rigorous experimental studies should examine which features of AI, users, and context lead to better coaching outcomes. Further, while AI educational coaches show positive associations with motivation (Fidan & Gencel, 2022; W. Huang et al., 2025; M. Liu & Reinders, 2025), further evaluations must be conducted to test for effects on overall performance to confirm that engagement translates into meaningful goal progress. Given that most of the literature relies heavily on self-reported data, these findings would benefit from other means of confirmation, such as by incorporating behavioral, physiological, and neural responses during these coaching sessions to better monitor the activities that AI encourages (Guan et al., 2025; W. Huang et al., 2025; Loughnane et al., 2025; Singh et al., 2023).

## 9. Conclusions

This narrative review sought to synthesize findings from empirical studies on purpose-built AI coaching chatbots in educational and health domains, discuss limitations, consider the risks of unsupervised use of general-purpose LLMs for coaching purposes, and reflect on implications for future research. Findings suggest that while some studies showed that purpose-built AI chatbots can support some coaching capabilities, such as promoting short-term motivation, self-reflection, and selected goal-directed behaviors, findings were mixed for other studies with very little evidence for long-term benefits or that AI chatbots were as effective as human coaches. With very little direct empirical evidence for the general public using general-purpose LLMs to seek out advice in these domains, we reflected on limitations and features of general-purpose LLMs that may pose particular risks, such as excessive sycophancy, “hallucinations,” and dependence, which may hinder personal development. As AI systems continue to evolve rapidly without much regulatory oversight, and as more people turn to them for everyday advice and guidance, more empirical, especially longitudinal, research is needed to determine how this new technology can best support personal development initiatives while minimizing harm. Since AI systems may be well-suited to serve as a complement to human coaching, future research should directly compare human-only, AI-only, and hybrid models longitudinally and for a variety of outcomes to see how AI could effectively aid the coaching process involving human oversight. Future research should also compare different AI systems (e.g., purpose-built vs. general-purpose) and examine which features of AI (e.g., availability, responsiveness, sycophancy, “hallucinations,” etc.) may be associated with potential benefits vs. risks. Such evidence is needed before stronger conclusions can be drawn about whether AI coaching may be able to support processes relevant to personal development and personal growth.

## Figures and Tables

**Table 1:** Working Classification of Chatbot and Language-Model Technologies Used in This Review.

Chatbot Technology	Approximate Introduction	Main Characteristic	Example Use in Coaching
Rule-based programming	1960s (e.g., Eliza in 1966)	Fixed rules and scripted responses	Simple coaching scripts
Task-oriented NLP/NLU chatbots	1970s–1980s	Classifies user intent within predefined domains	Purpose-built coaching chatbots
ML-Enhanced NLP/NLU chatbots	1990s–2000s	Learns patterns from training data to improve intent recognition	More adaptive or personalized purpose-built coaching systems
Early Large Language Models (LLMs)	Late 2010s–2022	Generate language from large text corpora but initially had limited broad public availability, scale, and conversational capability	Predominantly research-stage conversational system
Mass Market General-Purpose LLMs	Late 2022–Present	Generate open-ended responses across multiple domains without being restricted to predefined user intents	General-purpose conversational systems used informally for coaching (e.g., ChatGPT, Claude, Gemini)

*Note.* NLP = natural language processing; NLU = natural language understanding; ML = machine learning; LLM = large language model. NLP is the broader field concerned with enabling computational systems to process and generate human language, whereas NLU refers more specifically to interpreting meaning and intent. The categories are illustrative rather than mutually exclusive because a single system may combine scripted rules, NLP, NLU, machine-learning, and LLM-based components. The periods indicate when these approaches became more broadly available rather than the dates of their earliest development.

**Table 2:** Summary of Evidence Reviewed in Educational and Health Settings.

Theme	Domain	Studies and Reviews Cited	Overall Pattern
User experience and engagement	Education	(Chuang et al., 2023; Sia et al., 2021; Yin et al., 2024; Jasin et al., 2023; Neumann et al., 2021, 2025; Fryer et al., 2017)	Generally positive initial engagement and perceptions, although some studies reported frustration, limitations in chatbot interaction, preferences for human support, or declining interest over time.
Educational performance	Education	(Fidan & Gencel, 2022; Cai et al., 2021; Han et al., 2022; Ait Baha et al., 2023; Xu et al., 2021)	Mixed; some studies reported improved or comparable learning outcomes, whereas others found limited, null, or inferior effects relative to comparison conditions.
Psychological outcomes and conversational processes	Health	(Aymerich-Franch & Ferrer, 2022; Shah et al., 2026)	Evidence remains preliminary; studies have examined conversational processes and self-reported experiences, but the cited research does not establish downstream psychological effects.
Health behavior change	Health	(Hassoon et al., 2021; Santini et al., 2023; To et al., 2021; Maher et al., 2020; Dhinagaran et al., 2021; Chew et al., 2024)	Generally promising findings for physical activity and dietary behaviors, although several studies used pilot, feasibility, or uncontrolled designs, and evidence for sustained effects remains limited.
Sustained or clinically meaningful outcomes	Health	(Daley et al., 2020; Stasinaki et al., 2021)	Mixed; an uncontrolled evaluation reported reductions in symptoms, whereas an RCT did not demonstrate a sustained reduction in BMI-SDS, although improvements were observed in some physical outcomes.
Review-level evidence	Education	(Guan et al., 2025; W. Huang et al., 2025)	Generally promising findings for motivation and self-regulated learning, although findings were heterogeneous and the evidence base was dominated by short-term and self-reported outcomes.
Review-level evidence	Health	(Oh et al., 2021; Milne-Ives et al., 2020; Fu et al., 2026)	Mixed; reviews generally identify promising effects, but findings vary substantially across behaviors, interventions, and study designs, with limitations in study quality and evidence for sustained effects.

*Note*. The Studies and Reviews Cited column lists the AI-based chatbot and conversational-agent studies and reviews discussed in Section 4 and Section 5. The Overall Pattern column provides a qualitative narrative synthesis of the findings and is intended to indicate the broad direction and limitations of the evidence rather than a formal rating of intervention effectiveness. Because the included studies vary substantially in intervention type, outcome measure, study design, sample, and follow-up period, findings should not be interpreted as directly comparable or as a quantitative estimate of effectiveness.

**Table 3:** Potential Coaching Value, Risks, and Safeguards Associated With General-Purpose LLMs Used for Coaching.

LLM Characteristic	Potential Coaching Value	Principal Risk	Suggested Safeguard
Continuous availability	Immediate, on-demand prompts, guidance, and support	Frequent use, overreliance, or psychological dependence, particularly among lonely or distressed users	Preserve user agency, encourage appropriate limits on use, and clarify that the system is not a substitute for human or professional support
Personalized, responsive dialogue	Tailored reflection, encouragement, and metacognitive support	Anthropomorphism, misplaced trust, privacy concerns, and disclosure of sensitive information	Explain system limitations and data practices, protect privacy, and maintain appropriate human oversight
Agreeable, validating responses	Short-term satisfaction, perceived validation, and engagement	Sycophancy that reinforces existing beliefs or maladaptive thinking rather than providing constructive challenge	Evaluate and reduce sycophancy and redirect responses that reinforce distressing or maladaptive content
Open-ended generation and broad instruction-following	Flexible, on-demand information and generation of possible options across many goals	Hallucinated or inconsistent information and inappropriate responses to illogical or high-risk requests	Communicate uncertainty, encourage verification of consequential information, restrict high-risk guidance, and provide referral or escalation to qualified human support
Emotionally engaging interaction	Perceived warmth, responsiveness, and ease of disclosure	Emotional attachment, psychological dependence, and possible displacement of human or professional support	Establish clear boundaries between coaching, therapy, and crisis support and refer users to qualified professionals when appropriate

*Note.* LLM = large language model. The table presents an integrative synthesis of characteristics discussed across Section 3, Section 7 and Section 8. The stated coaching values are potential rather than consistently demonstrated benefits. Risks are supported by a combination of empirical research on general-purpose LLMs and adjacent research on social chatbots, together with conceptual, clinical, and governance sources. The safeguards are proposed measures and have not necessarily been tested empirically.

## Data Availability

No new data were created or analyzed in this study. Data sharing is not applicable to this article.

## Note

1 For a more detailed account of the history, classification, and architecture of chatbot technologies, see Adamopoulou and Moussiades (2020). For a more extensive review of the development, technical foundations, adaptation, and evaluation of LLMs, see Zhao et al. (2026).

## Supplementary Materials

The following supporting information can be downloaded at: https://www.mdpi.com/article/10.3390/bs16081431/s1, Table S1: Summary of primary studies reviewed in Section 4 (Educational) and Section 5 (Health).

## Abbreviations

The following abbreviations are used in this manuscript:

AI	Artificial Intelligence
NLP	Natural Language Processing
NLU	Natural Language Understanding
LLM	Large Language Model
