# Supplementary material for: Artificial Intelligence as a Personal Coach: A Narrative Review of Benefits and Risks in Educational and Health Contexts
Source: Behav Sci (Basel). 2026 Aug 19;16(8):1431. doi: 10.3390/bs16081431 (PMC13509547; doi:10.3390/bs16081431)
Supplement: Supplementary file 1 [file behavsci-16-01431-s001.zip › behavsci-4351917-supplementary.pdf]

Supplementary Table

Artificial Intelligence as a Personal Coach: A Narrative Review of Benefits and Risks in Educational and Health Contexts

Table S1 provides a study-by-study summary of the selected primary empirical AI-coaching studies discussed in Sections 4 and 5. It reports the type of AI system involved, the study design and duration, the broad type of outcome assessment, and the principal outcomes and findings. Review articles and non-AI studies are not included.

Table S1: Summary of primary studies reviewed in Sections 4 (Educational) and 5 (Health)

**Study**	**AI Type**	**Design & Duration**	**Outcome Assessment**	**Outcomes and Main Finding**
**Educational Domain**				
Ait Baha et al., 2023	ML/rules-based hybrid NLU chatbot	Three-group quasi-experimental classroom comparison; five 2-hour sessions	Objective + self-report	Slight gain vs. traditional teaching; best result was non-AI digital tools; chatbot improved reported engagement and learning experience
Cai et al., 2021	Rules-based NLU chatbot	Three online experiments; single-session lessons	Objective + self-report	Comparable learning gains to Khan Academy; ~40% preferred the chatbot
Chuang et al., 2023	Limited-AI chatbot + augmented reality	Cross-sectional post-use survey; duration not reported	Self-report	More enjoyable and engaging learning experience
Fidan & Gencel, 2022	ML/rules-based hybrid NLU chatbot	Three-group pretest-posttest quasi-experiment; 5 weeks	Objective + self-report	Improved performance and intrinsic motivation; no significant advantage over peer feedback
Fryer et al., 2017	Retrieval-based conversational AI chatbot	Counterbalanced experimental comparison with human partners during a 12-week English language course	Self-report	Chatbot task interest declined after the first interaction, consistent with a novelty effect; unlike human partner interactions, chatbot task interest did not predict later course interest

**Study**	**AI Type**	**Design & Duration**	**Outcome Assessment**	**Outcomes and Main Finding**
Han et al., 2022	Rules-based NLU chatbot	Non-equivalent-control pretest-post-test quasi-experiment; single-session intervention with immediate post-test	Objective + self-report	No significant improvement in objective learning outcomes; higher self-reported interest in education and self-directed learning vs. control
Jasin et al., 2023	ML/rules-based hybrid NLU chatbot	Mixed-methods pilot evaluation; 1 month	Self-report / qualitative	Mixed: increased confidence and self-directed learning when responses were helpful; frustration when hints were wrong
Neumann et al., 2021	ML/rules-based hybrid NLU chatbot	Implementation study with qualitative usability evaluations; chatbot deployed approximately 1 year	Self-report	Mixed reactions; students generally valued chatbot support, but some requested access to human staff/mentors
Neumann et al., 2025	Purpose-built chatbot built on a general-purpose LLM	Classroom deployment with post-use TAM survey and response accuracy audit; duration not clearly reported	Self-report + objective chatbot evaluation	High perceived usefulness and ease of use; students viewed it as a useful supplement but still preferred human tutors
Sia et al., 2021	ML/rules-based hybrid NLU chatbot	Single-group pre-post pilot; 1 week	Self-report	Modest short-term improvements in physical, social, and academic well-being
Xu et al., 2021	ML/rules-based hybrid NLU chatbot	Randomized two-group experiment; single story-reading session	Objective	Equivalent story comprehension to human partner; lower productivity, lexical diversity, and topical relevance, but higher intelligibility
Yin et al., 2024	Basic rule-based conversational coach	Randomized between-subjects experiment; single session	Self-report	Mixed emotional response (but primarily positive)

Study	AI Type	Design & Duration	Outcome Assessment	Outcomes and Main Finding
**Health Domain**				
Aymerich-Franch & Ferrer, 2022	Scripted, rule-based voice conversational agent (speech recognition + text-to-speech)	Uncontrolled pre-post coaching intervention; 3 weekly sessions over 3 weeks	Self-report	Increased personal growth initiative and life satisfaction; reduced negative affect
Chew et al., 2024	Basic rule-based digital coach	Single-group mixed-methods pre-post pilot evaluation; 1 week	Self-report	Improvements in overeating, snacking behavior, self-regulation, depressive symptoms, and physical activity
Daley et al., 2020	Rules-based NLU chatbot	Uncontrolled pre-post observational evaluation; 1 month	Self-report (clinical scales)	Reduced stress/anxiety/depression; ~half of participants initially above anxiety and depression cutoffs scored below these thresholds after use
Dhinagaran et al., 2021	Basic rule-based digital coach	Single-group web-based feasibility pilot; 4 weeks	Self-report	Small descriptive improvements in dietary behaviors (slightly increased fruit intake and reduced sweetened beverages/fried foods); no definitive behavioral improvements
Hassoon et al., 2021	Limited NLP/NLU conversational AI	Three-arm randomized controlled pilot trial; 5 weeks (1-week baseline + 4-week intervention)	Objective (tracked activity)	Voice-assisted AI coaching increased physical activity compared with control group
Maher et al., 2020	ML/rules-based hybrid NLU chatbot	Single-arm pre-post proof-of-concept study; 12 weeks	Objective + self-report	+110 min/wk activity; Mediterranean diet score rose 3.8 → 9.6
Santini et al., 2023	Basic rule-based digital coach	Mixed-methods single-group field trial; 10 weeks	Self-report	Increased physical activity; modest short-term gains in mental well-being/self-efficacy during human-supported use, but effects were not sustained after autonomous use

Study	AI Type	Design & Duration	Outcome Assessment	Outcomes and Main Finding
Shah et al., 2026	Purpose-built chatbot on general-purpose LLM	Longitudinal quantitative content analysis of chatbot dialogue; 1 week	Behavioral (coded dialogue)	User agency and autonomy behaviors were expressed by 97.4% of participants
Stasinaki et al., 2021	Chatbot-based digital coach (MobileCoach platform)	Two-group randomized controlled trial; 12 months	Objective/clinical	Improved physical capacity and body composition comparable to human-led behavioral intervention; no sustained BMI-SDS reduction
To et al., 2021	ML/rules-based hybrid NLU chatbot	Uncontrolled pre-post quasi-experiment; 6 weeks	Objective (tracked activity) + self-report	Self-reported physical activity increased by ~2.5 hours/week, with a smaller but significant increase in objectively measured Fitbit step counts

Note. AI = artificial intelligence; LLM = large language model; ML = machine learning; NLP = natural language processing; NLU = natural language understanding; TAM = technology acceptance model. The table lists studies by domain in alphabetical order of the first author. All systems listed were purpose-built or deployed for a specific educational, health, or coaching task. Neumann et al. (2025) and Shah et al. (2026) used purpose-built applications built on general-purpose LLMs; none examined unsupervised public use of a general-purpose LLM. Review articles and non-AI comparator studies are omitted.
